# Novel Biomarkers Associated With Progression and Prognosis of Bladder Cancer Identified by Co-expression Analysis

**DOI:** 10.3389/fonc.2019.01030

**Published:** 2019-10-11

**Authors:** Yejinpeng Wang, Liang Chen, Lingao Ju, Kaiyu Qian, Xuefeng Liu, Xinghuan Wang, Yu Xiao

**Affiliations:** ^1^Department of Urology, Zhongnan Hospital of Wuhan University, Wuhan, China; ^2^Department of Biological Repositories, Zhongnan Hospital of Wuhan University, Wuhan, China; ^3^Human Genetics Resource Preservation Center of Hubei Province, Wuhan, China; ^4^Human Genetics Resource Preservation Center of Wuhan University, Wuhan, China; ^5^Department of Pathology, Lombardi Comprehensive Cancer Center, Georgetown University Medical School, Washington, DC, United States; ^6^Laboratory of Urology, Medical Research Institute, Wuhan University, Wuhan, China; ^7^Laboratory of Precision Medicine, Zhongnan Hospital of Wuhan University, Wuhan, China

**Keywords:** bladder cancer (BC), gene-set enrichment analysis (GSEA), protein-protein interaction (PPI), weighted co-expression network analysis (WGCNA), The Cancer Genome Atlas (TCGA) dataset

## Abstract

Our study's goal was to screen novel biomarkers that could accurately predict the progression and prognosis of bladder cancer (BC). Firstly, we used the Gene Expression Omnibus (GEO) dataset GSE37815 to screen differentially expressed genes (DEGs). Secondly, we used the DEGs to construct a co-expression network by weighted gene co-expression network analysis (WGCNA) in GSE71576. We then screened the brown module, which was significantly correlated with the histologic grade (*r* = 0.85, *p* = 1e-12) of BC. We conducted functional annotation on all genes of the brown module and found that the genes of the brown module were mainly significantly enriched in “cell cycle” correlation pathways. Next, we screened out two real hub genes (ANLN, HMMR) by combining WGCNA, protein-protein interaction (PPI) network and survival analysis. Finally, we combined the GEO datasets (GSE13507, GSE37815, GSE31684, GSE71576). Oncomine, Human Protein Atlas (HPA), and The Cancer Genome Atlas (TCGA) dataset to confirm the predict value of the real hub genes for BC progression and prognosis. A gene-set enrichment analysis (GSEA) revealed that the real hub genes were mainly enriched in “bladder cancer” and “cell cycle” pathways. A survival analysis showed that they were of great significance in predicting the prognosis of BC. In summary, our study screened and confirmed that two biomarkers could accurately predict the progression and prognosis of BC, which is of great significance for both stratification therapy and the mechanism study of BC.

## Introduction

BC is one of the most common malignancies of the urinary tract (1), and is a complex disease with high morbidity and mortality if not diagnosed timely and treated optimally (2). It is estimated that there are 429,000 new cases and 165,000 deaths worldwide each year (3). The most common symptom of BC is painless hematuria, which is seen in more than 80% of patients. At present, BC can be divided into two major categories according to tumor stage: non-muscle invasive bladder cancer (NMIBC) and muscle-invasive bladder cancer (MIBC) (4, 5). NMIBC is characterized by the co-activation of FGFR3 mutations, high recurrence rate (50–70%), and the 5-year survival rate > 90% (6). However, MIBC is characterized by frequent TP53 mutations, high metastasis and a 5-year survival rate <50% (7). 70–80% of BC patients had non-muscle-invasive bladder cancer (NMIBC) (8), and 20–30% of these patients will progress to MIBC (9). Once BC progression is detected, the patient's prognosis decreases (10, 11); currently, there is a lack of effective biomarkers that can accurately predict the progress and prognosis of BC, so such biomarkers need to be discovered urgently.

With the rapid development of microarray and high-throughput sequencing technology, bioinformatics plays an important role in various fields (12–15). In the medical field, the most commonly used means of bioinformatics is to find biomarkers (16–18). However, at present, many studies only consider the differences in gene expression between different samples, and only look for biomarkers with differential expression as the limiting condition, while ignoring the underlying connection of each gene (19, 20).

Here, we constructed WGCNA co-expression network and incorporated genes with similar expression patterns into the same modules. After all the modules were related to the calculation of clinical phenotype data, the modules most related to the progression of BC were obtained. Finally, after a series of screening tests, we found the real hub genes (ANLN, HMMR) that could truly predict the progression and prognosis of BC. Our study fully considered the internal relationship between genes, rather than only considering differential expression genes. The GSEA analysis and functional annotation showed that the real hub genes played their role in BC through signaling pathways such as “bladder cancer” and “cell cycle.” We combined a large number of databases (GEO, TCGA, Oncomine, HPA, String, GEPIA, GSCALite) to verify the ability of real hub genes to predict the progression and prognosis of BC, ensuring the stability and reliability of the results.

## Materials and Methods

### Data Collection and Study Design

**Figure 1:**
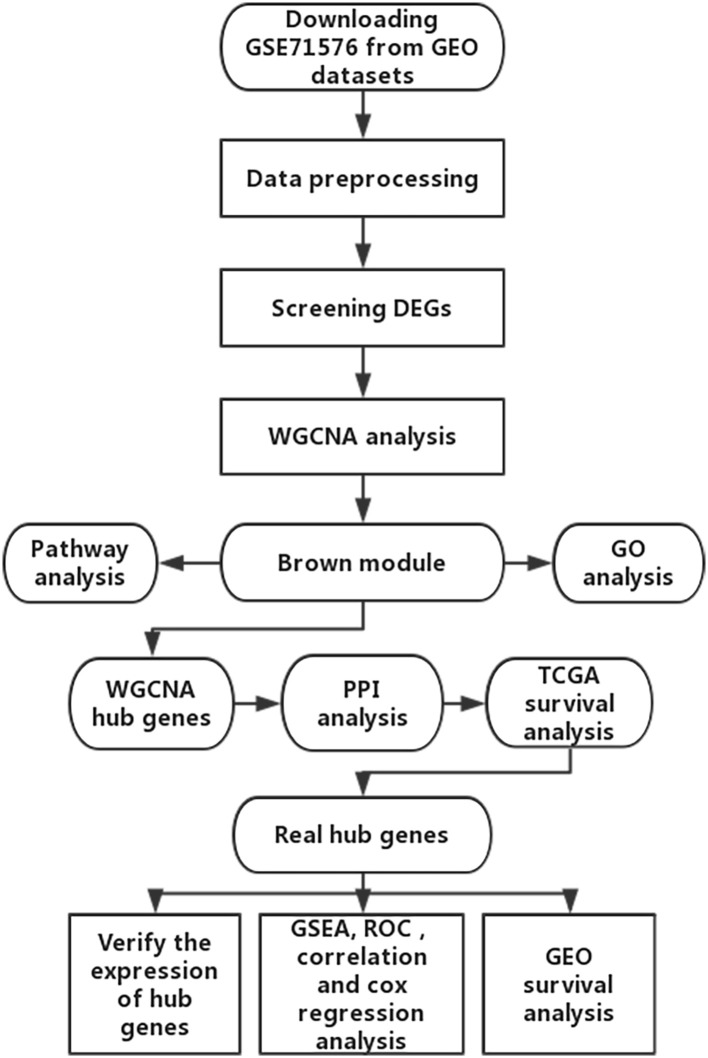
Flow diagram of the study.

The microarray dataset GSE13507, GSE31684, GSE37815, GSE71576 and the corresponding clinical information data of these microarray datasets were downloaded from the Gene Expression Omnibus (GEO) database of the NCBI database (https://www.ncbi.nlm.nih.gov/). The datasets GSE37815 and GSE13507 both performed on the Illumina human-6 v2.0 platform, the former was used to screen for different expression genes (DEGs), the latter was used to verify the hub genes. The dataset GSE71576, which performed on the Affymetrix Human Gene 1.0 ST platform, was used to perform weighted co-expression network analysis. The dataset GSE31684, which performed on the Affymetrix Human Genome U133 Plus 2.0 platform, was also used to verify the hub genes. The level three RNA-seq data (Illumina RNASeqV2) and corresponding clinical information about BC were downloaded from The Cancer Genome Atlas (TCGA) database (http://cancergenome.nih.gov/). The dataset, which included 408 BC samples and 19 normal bladder samples, was used to verify the hub genes, perform GSEA, correlation analysis and survival analysis. The inclusion cohort was defined as a cohort containing microarray or RNA-seq data and clinical phenotypes and follow-up data. By consulting the literature, we took the cohorts without performed WGCNA as training sets and internal validation sets, and the cohorts that have undergone WGCNA research as external validation sets. Dataset GSE37815 contained 18 BC and 6 normal bladder samples, so we chose it for DEGs analysis. Furthermore, we chose datasets GSE37815 and GSE71576 as training and internal validation datasets, whereas the datasets GSE13507, GSE31684, and TCGA were set as external validation datasets. The detailed information of these datasets was listed in Table 1, and the flow chart of our entire experiment is presented in Figure 1.

**Table 1:** Information of datasets used in this study.

**Datasets**	**GSE37815**	**GSE71576**	**GSE13507**	**GSE31684**	**TCGA**
	**Training validation datasets**		**External validation datasets**		
**Platform**	**Illumina human-6 v2.0**	**Affymetrix human gene 1.0 ST**	**Illumina human-6 v2.0**	**Affymetrix human genome U133 plus 2.0**	**Illumina RNASeqV2**
**SAMPLE NUMBER**					
Total	18	44	256	93	427
Bladder cancer	6	44	165	93	408
Normal bladder	–	0	68	0	19
Recurrent bladder cancer	–	–	23	–	–
pStage I	–	–	–	10	2
pStageII	–	–	–	17	130
pStage III	–	–	–	42	140
pStage IV	–	–	–	19	135
Unknown stage	–	–	0	5	1
Grade I	–	14	105	–	–
GradeII	–	11	60	–	–
Grade III	–	17	0	–	–
High grade	–	–	–	87	385
Low grade	–	–	–	6	22
Unknown grade	–	2	–	–	1
Ta	–	27	24	–	–
T1	–	6	80	–	–
T2	–	3	31	–	–
T3	–	2	19	–	–
T4	–	4	11	–	–
Unknown T stage	–	2	–	–	–

### Data Preprocessing and DEGs Screening

All the raw expression data were subject to quality control, background correction, normalization, logarithmic conversion and remove batch effects processing, using the R packages “affy” (21) or “limma” (22). After that, samples without clinical data were filtered out, and the resulting data were subsequently analyzed. The RNA-seq data of the TCGA dataset were normalized using the “DESeq2” (23) R package. The “limma” R package was used to screen the DEGs between eighteen BC and six normal bladder samples in dataset GSE37815. The false discovery rate (FDR) <0.05 and |log2FC| ≥1 were set as the threshold for screening DEGs.

### Establishment of Weighted Co-expression Network

The DEGs were used to construct a weighted co-expression network by the R package “WGCNA” (24). Firstly, we used the function “goodSamplesGenes” in the “WGCNA” package checked to see if the input genes (DEGs) and input samples were good genes and good samples. Secondly, Pearson's correlation analysis of all pairs of genes was used to construct an adjacency matrix. After that, the adjacency matrix was used to construct a scale-free co-expression network based on a soft-thresholding parameter β (β was a soft-thresholding parameter that could enhance strong correlations between genes and penalize weak correlations) (25). The adjacency matrix was then turned into a topological overlap matrix (TOM). TOM could measure the network connectivity of a gene, which was defined as the sum of its adjacency with all other genes, and was used for network generation (26). At the same time, in order to classify genes with similar expression patterns into gene modules, average linkage hierarchical clustering was conducted according to the TOM-based dissimilarity measure with a minimum size (gene group) of 50 for the genes dendrogram.

### Identify Significant Relevant Module and Module Functional Annotation

To investigate the biological function of the brown module, which significantly related to the histologic grade of BC, we uploaded the list of all genes in the brown module to the DAVID website (https://david.ncifcrf.gov) for functional annotation analysis. The threshold was the *p* < 0.05.

### Real Hub Genes Identification by WGCNA, PPI, and Survival Analysis

By calculating the correlation between modules and clinical phenotypes by the module-trait relationship of WGCNA, we could screen the module most relevant to the clinical phenotype we were interested in. In our study, histologic grade (*r* = 0.85, *p* = 1e-12) was selected as interested clinical phenotype for subsequent analysis.

After the interesting module was chosen, same as in the past (27, 28), we defined the cor.geneModuleMembership > 0.8 (the correlation between the gene and a certain clinical phenotype) and cor.geneTraitSignificance > 0.2 (the correlation between the module eigengene and the gene expression profile) as the threshold for screening hub genes in a module.

To further target and screen more meaningful hub genes, we uploaded the list of 49 hub genes to the STRING database (https://string-db.org/) to construct a protein-protein interaction (PPI) network (29). The minimum interaction score of these genes was >0.4 and were defined as the threshold of the hub genes of the PPI network. The Cytoscape software (30) was used to visualize network diagrams for PPI analysis. Finally, we used the Gene Expression Profiling Interactive Analysis (GEPIA) database (31) (http://gepia.cancer-pku.cn/) to test the prognostic value of hub genes, and the hub genes with the ability to predict prognosis were the real hub genes. To verify the value of predicting prognosis of hub genes, a survival analysis of real hub genes was performed using the GSE13507 dataset from GEO datasets.

### Gene Set Enrichment Analysis of Real Hub Genes

The GSEA software was downloaded from http://software.broadinstitute.org/gsea/index.jsp. The GSEA analysis was conducted with a small cohort GSE37815 and a large cohort TCGA dataset, respectively. We divided the samples into two groups according to the median expression of hub genes, and chose the C2 (c2.cp.kegg.v6.1.symbols.gmt) sub-collection downloaded from the Molecular Signatures Database (http://software.broadinstitute.org/gsea/msigdb/index.jsp) as the reference gene sets to perform GSEA analysis.

### Verify the Expression Pattern and the Prognostic Value of Real Hub Genes

The datasets GSE37815 and GSE71576 were selected as internal validation datasets, the datasets GSE31684, GSE13507, and TCGA were set as external validation datasets. All of them were used to verify the real hub genes' mRNA expression pattern in different histologic grades or pathologic stage of BC. In addition, we used the Oncomine database (https://www.oncomine.org/resource/main.html) and the above dataset to verify the expression of real hub genes between BC tissues and adjacent tissues. We used the one-way analysis of variance (ANOVA) or Student's *t*-test to measure the statistical significance of the calculated results. After that, we performed a Kaplan-Meier survival analysis of hub genes in each cohort using the “survival” R package.

## Results

### Screening of Differentially Expressed Genes

The R package “limma” was used to screen DEGs between BC and normal bladder samples in GSE37815, where a total of 792 DEGs were screened (240 up-regulated and 552 down-regulated) under the threshold of FDR <0.05 and logFC (fold change) ≥1. The heatmap of DEGs is shown in Supplementary Figure S1, and all DEGs are listed in Supplementary Table S1.

### Establishment of Co-expression Network

**Figure 2:**
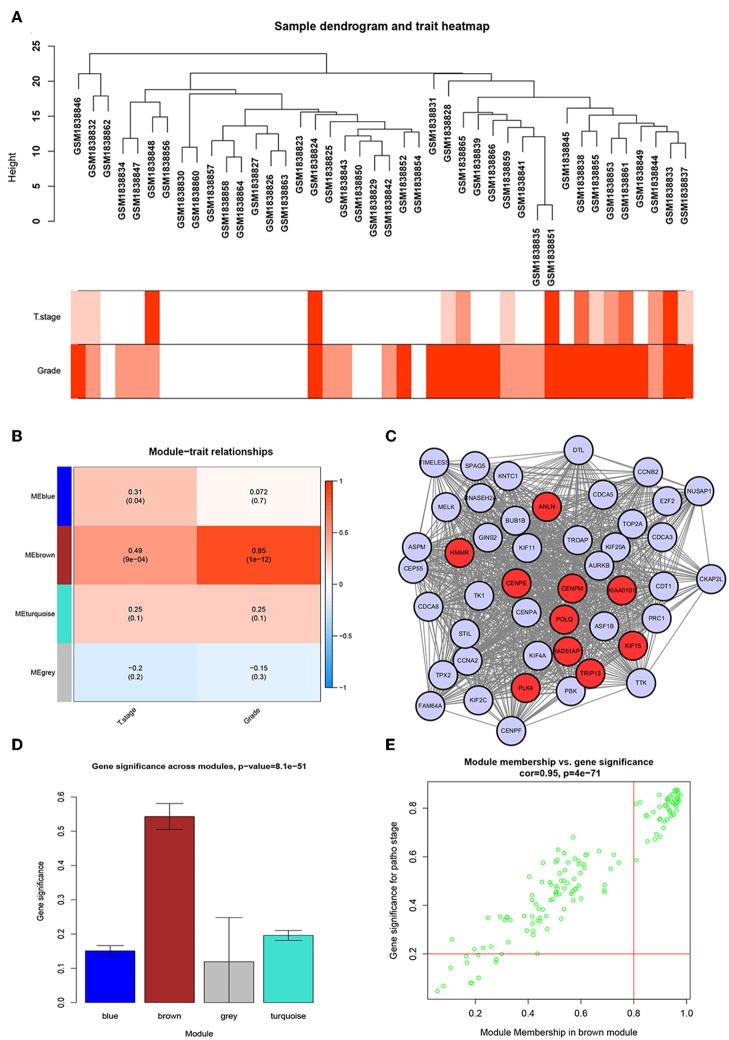
WGCNA and PPI network analysis. **(A)** Sample dendrogram and trait indicator. The clustering was a visual result of calculations based on Pearson correlation coefficients between samples. The color intensity was proportional to T stage and histologic grade of BC. **(B)** Identification of modules associated with the clinical traits of BC. **(C)** PPI network of WGCNA hub genes, the red nodes represent the hub genes in the PPI network. **(D)** Distribution of average gene significance and errors in the modules associated with histologic grade of BC. **(E)** Scatter plot of module eigengenes related to histologic grade in the brown module.

We used the R package of “WGCNA” to construct the weighted co-expression network. No outlier samples were found by Pearson correlation analysis (Figure 2A). We put 792 DEGs with similar expression patterns into modules by cluster analysis. In this study, the power of β = 6 (scale-free *R*^2^ = 0.95) was chosen for the soft-thresholding to ensure a scale-free network (Supplementary Figures S2A–D), and we got four modules for the next analysis (Supplementary Figure S2E).

### Identification of the Most Significant Modules

To identify genes associated with the progression of BC, we analyzed the association between modules and clinical phenotypes. The modules most significantly associated with tumor grade and T stage are of great value in predicting BC progression. Histologic grade (*r* = 0.85, *p* = 1e-12) and T stage (*r* = 0.49, *p* = 9e-04, Figure 2B) were significantly associated with brown module by Module-feature relationship analysis. Besides, the brown module had the highest gene significance in relation to histologic grade (Figure 2D). Therefore, we chose the brown module for further analysis.

### Brown Module Functional Annotation

**Figure 3:**
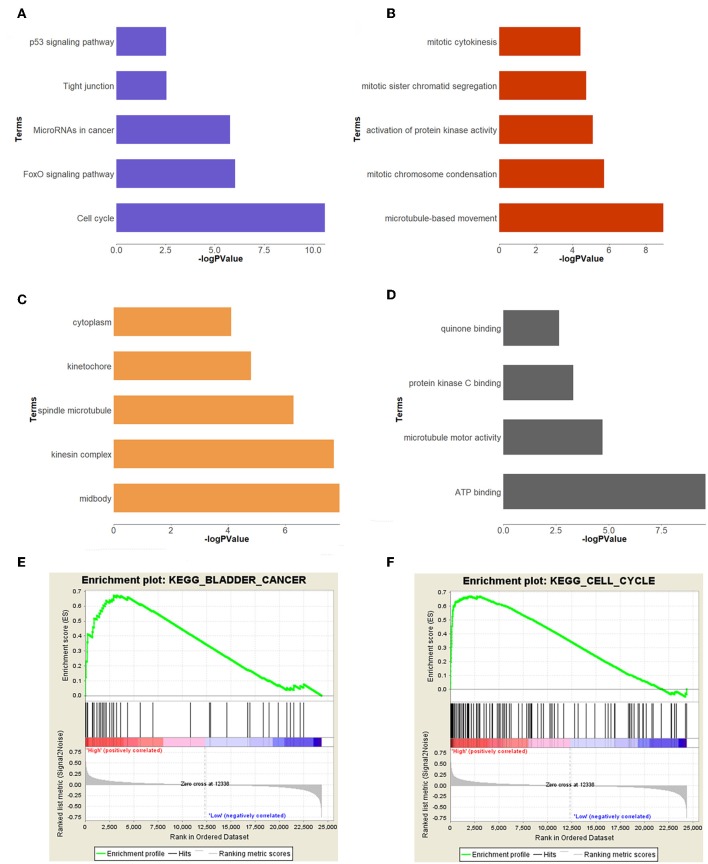
Functional annotation and GSEA analysis for brown module. **(A)** The signaling pathways, **(B)** biological process, **(C)** cellular components, **(D)** molecular composition of the brown module. **(E,F)** GSEA analysis revealed that the genes of brown module were mainly enriched in bladder cancer and cell cycle related pathways.

In order to study the function of the brown module, we uploaded the list of all genes in the brown module to the DAVID (https://david.ncifcrf.gov) website for a functional annotation analysis. The KEGG analysis revealed that the “cell cycle,” “FoxO signaling pathway,” “Tight junction,” “MicroRNAs in cancer,” and “p53 signaling pathway” were mainly enriched in the brown module (Figure 3A). The biological process of the brown module was mainly related to “microtubule-based movement,” “mitotic chromosome condensation,” “activation of protein kinase activity,” and so on (Figure 3B). The cell component of brown module was mainly enriched in “midbody,” “kinesin complex,” “spindle microtubule,” etc. (Figure 3C). And the molecular function was mainly enriched in “ATP binding,” “microtubule motor activity,” “protein kinase C binding,” etc. (Figure 3D). The threshold was the *p* < 0.05. The information of functional annotation is listed in Supplementary Table S2.

### Identification of Real Hub Genes

**Figure 4:**
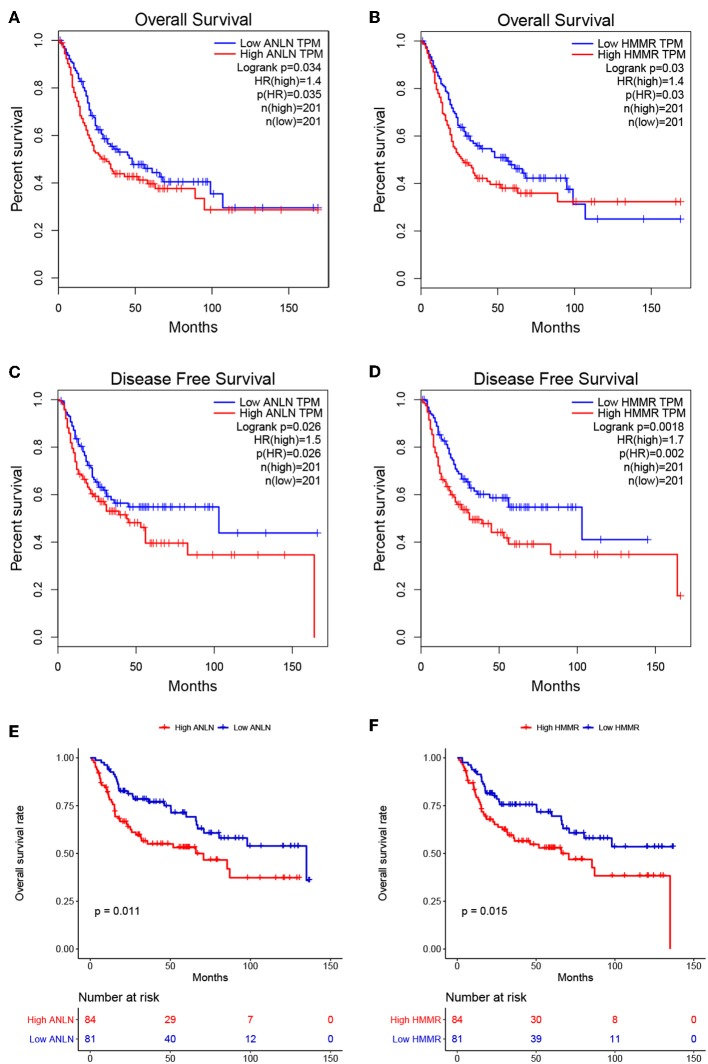
Survival analyses on real hub genes in the TCGA and GEO database. **(A,B)** Overall survival analysis related to ANLN **(A)** or HMMR **(B)** expression levels in the TCGA database. **(C,D)** Disease-free survival analyses related to ANLN **(C)** or HMMR **(D)** expression levels in the TCGA database. **(E,F)** Overall survival analysis related to ANLN **(E)** or HMMR **(F)** in the GEO database (GSE13507).

To further screen for the most significant hub genes, we combined three methods (WGCNA, PPI, and survival analysis) to screen real hub genes together. First, 49 hub genes with high connectivity were screened out from the brown module (Figure 2E). Secondly, we uploaded these 49 hub genes to the STRING database for a PPI network analysis. Under the threshold of a minimum required interaction score > 0.4, 10 hub PPI genes were screened (Figure 2C, Supplementary Table S3). Finally, we used the GEPIA database for the survival analysis of these 10 hub genes, and the hub genes with the ability to predict prognosis were real hub genes (ANLN, HMMR, Supplementary Table S4). The results showed that both real hub genes were predictive of overall survival and disease-free survival in BC (Figures 4A–D, Supplementary Table S3). Meanwhile, the external validation dataset GSE13507 was used to confirm the prognostic value of real hub genes (Figures 4E,F).

### GSEA Analysis of Real Hub Genes

In order to explore the functions and pathways of these two hub genes, we conducted GSEA on these hub genes, respectively. The GSEA analysis of two hub genes in the GSE37815 dataset revealed that the samples of highly expressed real hub genes were mainly enriched in “bladder cancer,” “cell cycle,” and “ubiquitin mediated proteolysis” related pathways (Figures 3E,F, Table 2). Subsequently, our GSEA analysis in the TCGA database produced similar results (Supplementary Tables S7–S9).

**Table 2:** Results of GSEA analysis based on the expression level of hub genes.

**Group**	**Term**	**Enrichment score**	**NOM *p*-val**
High ANLN/HMMR	KEGG_BLADDER_CANCER	0.674	0.004
	KEGG_CELL_CYCLE	0.671	0.010
	KEGG_UBIQUITIN_MEDIATED_PROTEOLYSIS	0.431	0.012
	KEGG_HOMOLOGOUS_RECOMBINATION	0.717	0.014
	KEGG_RNA_DEGRADATION	0.485	0.022
	KEGG_PROGESTERONE_MEDIATED_OOCYTE_MATURATION	0.495	0.026
	KEGG_BASE_EXCISION_REPAIR	0.643	0.032
	KEGG_MISMATCH_REPAIR	0.730	0.040
	KEGG_NUCLEOTIDE_EXCISION_REPAIR	0.613	0.045
Low ANLN/HMMR	KEGG_TYPE_II_DIABETES_MELLITUS	−0.475	0.004
	KEGG_NATURAL_KILLER_CELL_MEDIATED_CYTOTOXICITY	−0.520	0.014
	KEGG_T_CELL_RECEPTOR_SIGNALING_PATHWAY	−0.446	0.016
	KEGG_DILATED_CARDIOMYOPATHY	−0.618	0.018
	KEGG_HYPERTROPHIC_CARDIOMYOPATHY_HCM	−0.606	0.026
	KEGG_HEMATOPOIETIC_CELL_LINEAGE	−0.647	0.027
	KEGG_HISTIDINE_METABOLISM	−0.675	0.030
	KEGG_TRYPTOPHAN_METABOLISM	−0.643	0.034
	KEGG_FOCAL_ADHESION	−0.580	0.047

### Verification of the Expression Pattern of Real Hub Genes

Since these real hub genes were screened out by DEGs, we first verified the expression pattern of real hub genes between BC and paracancerous. The results showed that the expression of real hub genes was up-regulated in BC (Supplementary Figure S3), and the results were consistent in multiple datasets (Oncomine dataset, GSE13507, GSE37815, and TCGA dataset). Secondly, since the real hub genes belong to the brown module, which was significantly related to the histological grade and pathological stage of BC, the expression pattern of ANLN (Figure 6) and HMMR (Figure 7) in different histological grade and pathological stage were verified in internal validation datasets (GSE71576) and external validation datasets (GSE13507, GSE31684, and TCGA dataset). The one-way analysis of variance (ANOVA) or Student's *t*-test was used to measure the statistical significance of the calculated results. The results of receiver operating characteristic curve (ROC) analysis showed that real hub genes could well distinguish cancer and paracancer, different grades, different stages, NMIBC and MIBC (Supplementary Table S5). In addition, we verified the expression patterns of the protein levels of ANLN and HMMR in tissues in the HPA database, and found that the higher the grade of BC, the higher the protein levels of these two genes were (Supplementary Figure S6).

### Validation of Prognostic Value of Real Hub Genes

**Figure 5:**
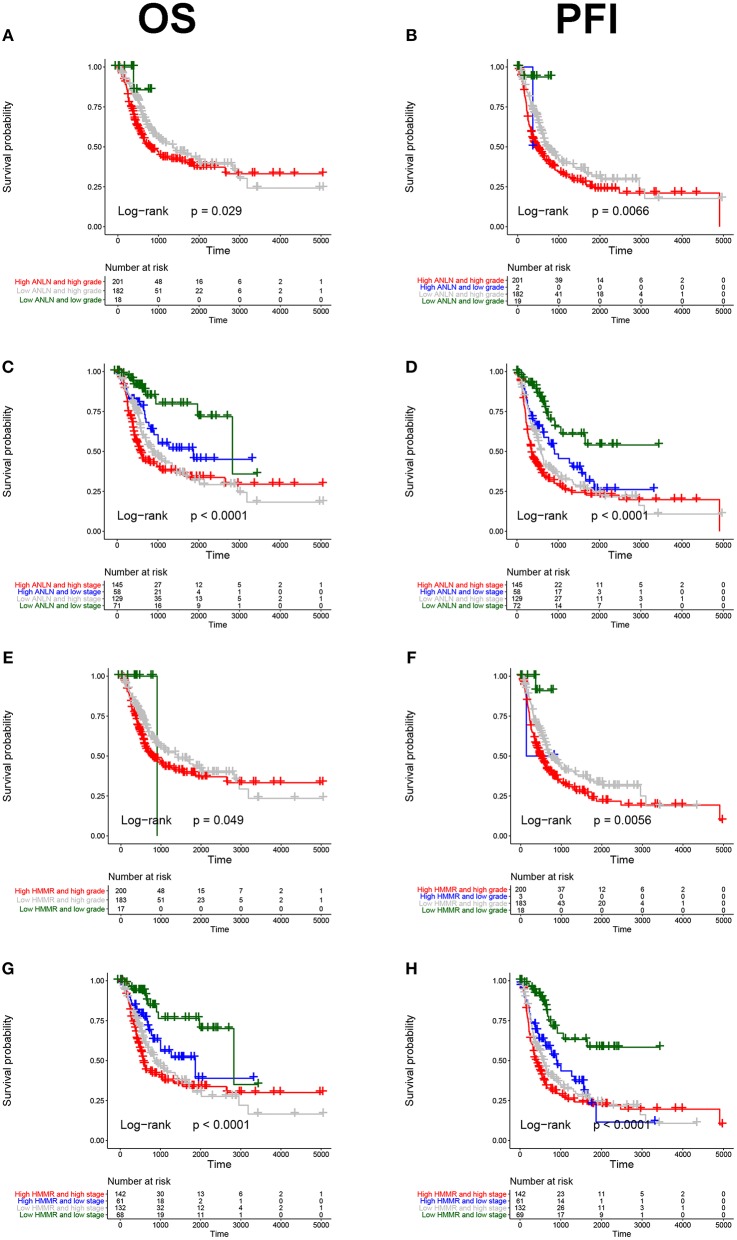
Subgroup survival analysis of hub genes. Overall survival (left panels) and Progression-free interval analyses (right panels) of ANLN were performed in different tumor grade subtypes **(A,B)**; Again, we did the same analysis in tumor stage subtypes **(C,D)**; Corresponding to the previous analysis, we did the same analysis for HMMR **(E–H)**.

To further explore the prognostic value of hub genes in BC, we conducted a subgroup survival analysis of these two genes in the TCGA dataset. The results showed that these two genes showed significant prognostic value in different stages and grades, which could not only accurately predict the overall survival rate of BC, but also predict its progression-free interval (PFI) event (Figure 5).

**Figure 6:**
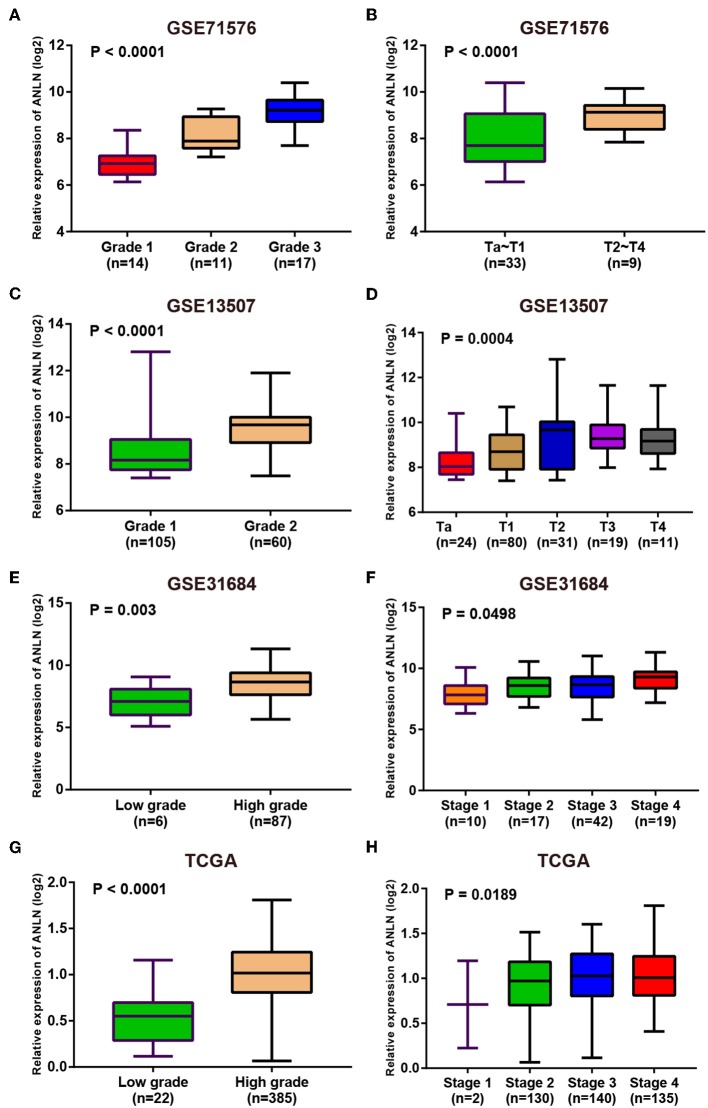
Expression pattern validation for ANLN. ANLN in GSE71576 **(A,B)** GSE13507 **(C,D)** GSE31684 **(E,F)**, TCGA database **(G,H)** of different grade and stage of expressing validation. Statistical differences in these data were calculated using One-way analysis of variance (ANOVA) or Student's *t*-test.

**Figure 7:**
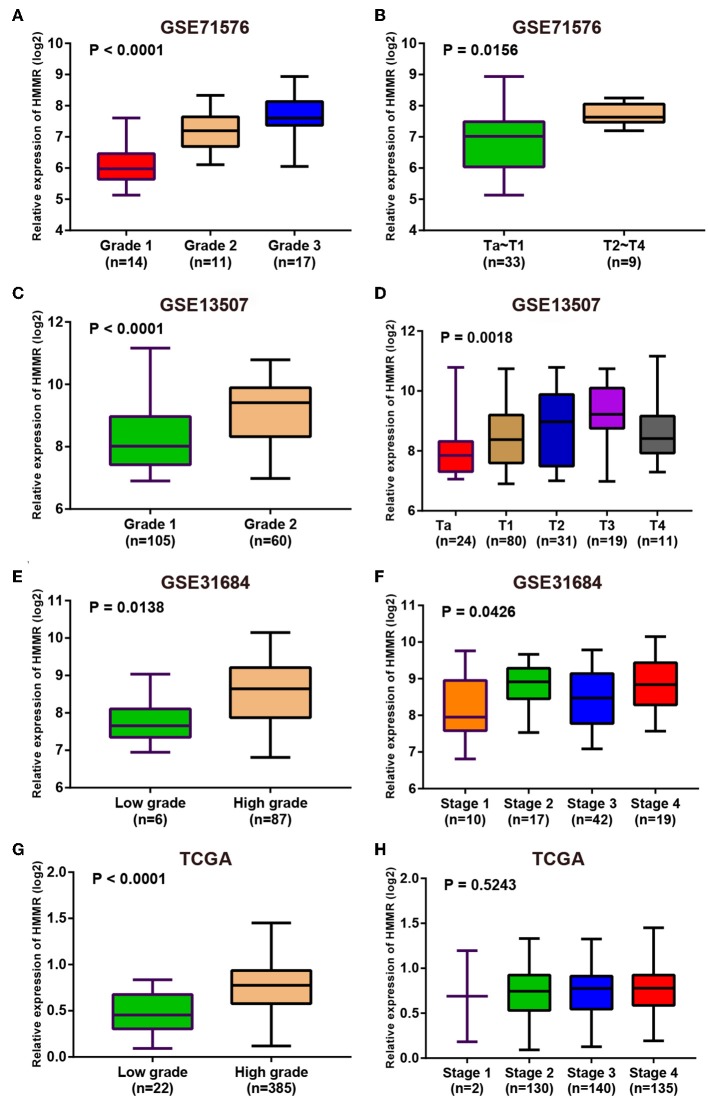
Expression pattern validation for HMMR. HMMR in GSE71576 **(A,B)** GSE13507 **(C,D)** GSE31684 **(E,F)**, TCGA database **(G,H)** of different grade and stage of expressing validation. Statistical differences in these data were calculated using One-way analysis of variance (ANOVA) or Student's *t*-test.

### Drug Sensitivity of Real Hub Genes

GSCALite (http://bioinfo.life.hust.edu.cn/web/GSCALite/) is a web-based analysis platform for gene set cancer analysis (32). We used this database to analyze drug sensitivity of real hub genes, which provides support for drug selection of real hub genes targeted therapy.

Finally, we explored the drug sensitivity of real hub genes using the GSCALite database, and the results were shown in Supplementary Figure S5, which provides support for drug targeted therapy of real hub genes.

## Discussion

BC is one of the most common tumors of the urinary system. Currently, radical cystectomy is the most effective treatment for BC, but in most cases, this treatment will greatly reduce the quality of life of patients (33). Therefore, it is urgent to find biomarkers that can accurately predict the progression and prognosis of BC.

Through a series of rigorous screening, two real hub genes (ANLN, HMMR) that could accurately predict the progression and prognosis of BC were found. Similar studies have focused mostly on one clinical phenotype (34–36). Our study conducted correlation analysis of T staging and grading as both clinical phenotypes and modules are of interest to us, and the results revealed that the brown module was highly correlated with both T staging (*r* = 0.49, *p* = 9e-04) and grading (*r* = 0.85, *p* = 1e-12). We then used a lot of datasets to verify this, and it turned out that the real hub genes were actually significantly correlated with BC T stage, pathological stage, and histological grade. Moreover, we also hoped to find biomarkers that could accurately predict the prognosis of BC, so we used survival analysis as a screening condition, which was neglected in some similar studies (37, 38). In the following two hub genes, we analyzed the survival analysis of the two hub genes and analyzed them in different subgroups (stage, grade), and found that these two genes had a high prognostic value for BC.

The excessive proliferation of tumors is often accompanied by cell cycle disorders. We used GSEA analysis to explore the function of real hub genes, and we found that both ANLN and HMMR were significantly enriched in functions and pathways related to “cell cycle.” Correlation analysis also supports this result. These two genes were also enriched in the pathway related to “bladder cancer,” and we speculate that these two genes may play a key role in the pathogenesis of BC.

ANLN (Anillin) is an actin-binding protein and has reportedly been shown to be significantly upregulated in the BC, knockdown of ANLN results in G2/M phase block and reduces expression of cyclin B1 and D1, and it was also demonstrated that ANLN can promote the progression, migration, and invasion of BC (39). Other studies have found that ANLN could promote the progression of pancreatic cancer by inducing the up-regulation of EZH2 by mediating the mir-218-5p /LASP1 signaling axis (40). ANLN has also been found to play a key role in the development of human lung cancer (41). All these suggest that ANLN plays a very important role in the development and progression of tumors. We found a high correlation between ANLN and CIRBP (Supplementary Figure S4B, Supplementary Table S6), a gene that we studied before (42); therefore, we can further explore the interaction between ANLN and CIRBP in the pathogenesis of BC. We also found a strong correlation between ANLN and KIF23 (Supplementary Figure S4A, Supplementary Table S6), an independent prognostic target for glioma (43).

HMMR (Hyaluronan Mediated Motility Receptor) is widely expressed in many types of tumors, including prostate and breast cancer, and various forms of leukemia (44–46). Previously reported overexpression of HMMR is associated with the development of metastatic prostate cancer (PCa) and castration-resistant PCa (46). But HMMR has never been studied in human BC, so our study found a new potential biomarker for BC. We found a strong correlation between HMMR and KIF20A (Supplementary Figure S4C, Supplementary Table S6), and a recent study found that KIF20A affects the prognosis of BC by promoting the proliferation and metastasis of BC (47). These studies are very helpful for our future research on the pathogenesis of HMMR in BC.

Taken together, through the integrated analysis of multiple databases and the establishment of the co-expression network by WGCNA analysis, two hub genes that can accurately predict the progression and prognosis of BC were screened out layer by layer, providing potential targets for the pathogenesis and treatment selection of BC.

## Data Availability Statement

Publicly available datasets were analyzed in this study. This data can be found here: https://www.ncbi.nlm.nih.gov/.

## Author Contributions

YW, XW, and YX conceived and designed the study. YW and LC performed the analysis procedures. YW, LC, LJ, KQ, XL, and YX analyzed the results. YW, LC, and YX contributed analysis tools. YW, LC, XW, and YX contributed to the writing of the manuscript. All authors reviewed the manuscript.

### Conflict of Interest

The authors declare that the research was conducted in the absence of any commercial or financial relationships that could be construed as a potential conflict of interest.
