# Supplementary material for: Novel Biomarkers Associated With Progression and Prognosis of Bladder Cancer Identified by Co-expression Analysis
Source: Front Oncol. 2019 Oct 11;9:1030. doi: 10.3389/fonc.2019.01030 (PMC6799077; doi:10.3389/fonc.2019.01030)
Supplement: Supplementary file 1 [file Data_Sheet_1.docx]

**Novel biomarkers associated with progression and prognosis of bladder cancer identified by co-expression analysis**

**Supplementary figures**


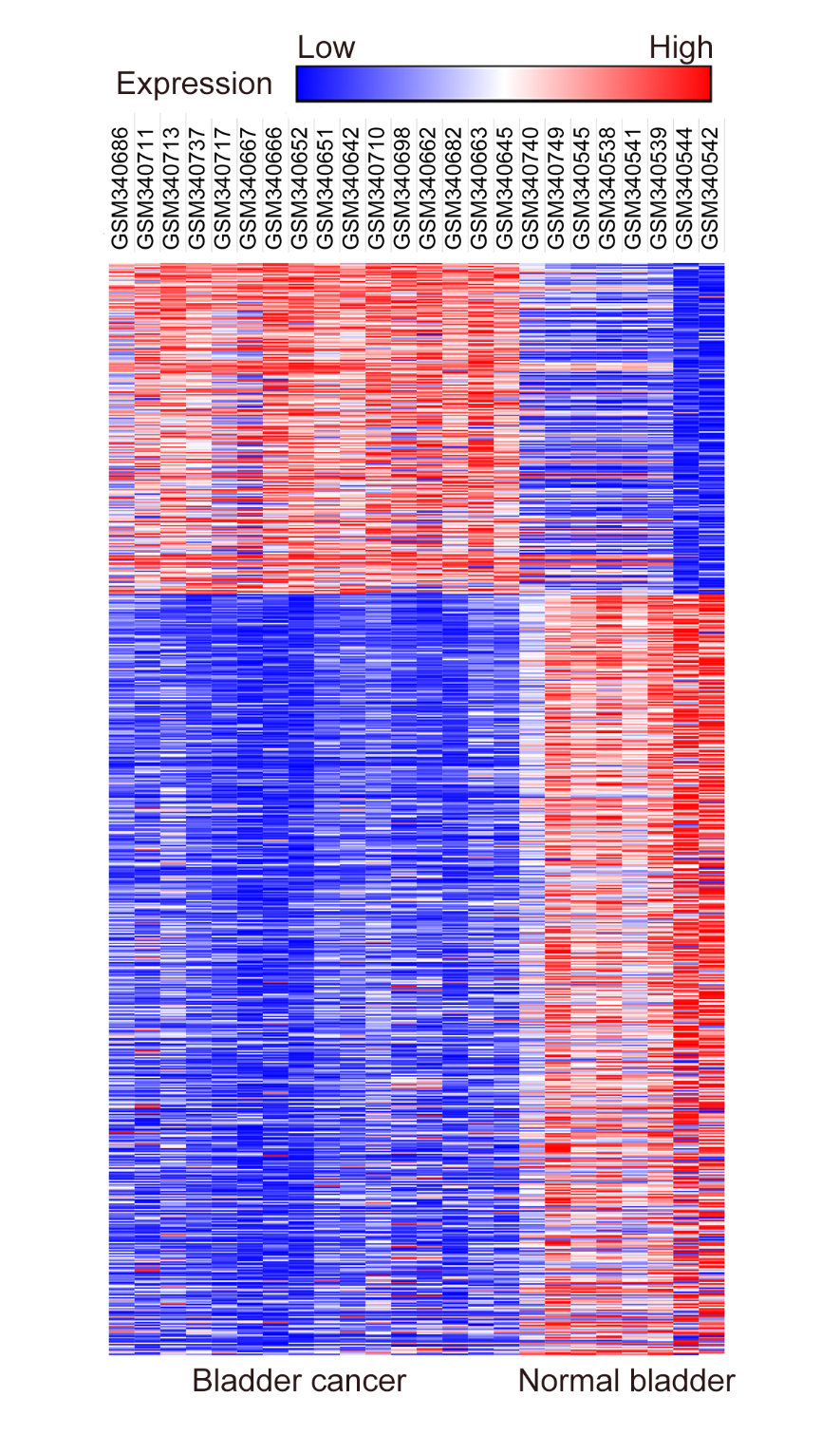


**Supplementary Figure S1.** Heatmap of the DEGs between bladder cancer and normal bladder (GSE37815).


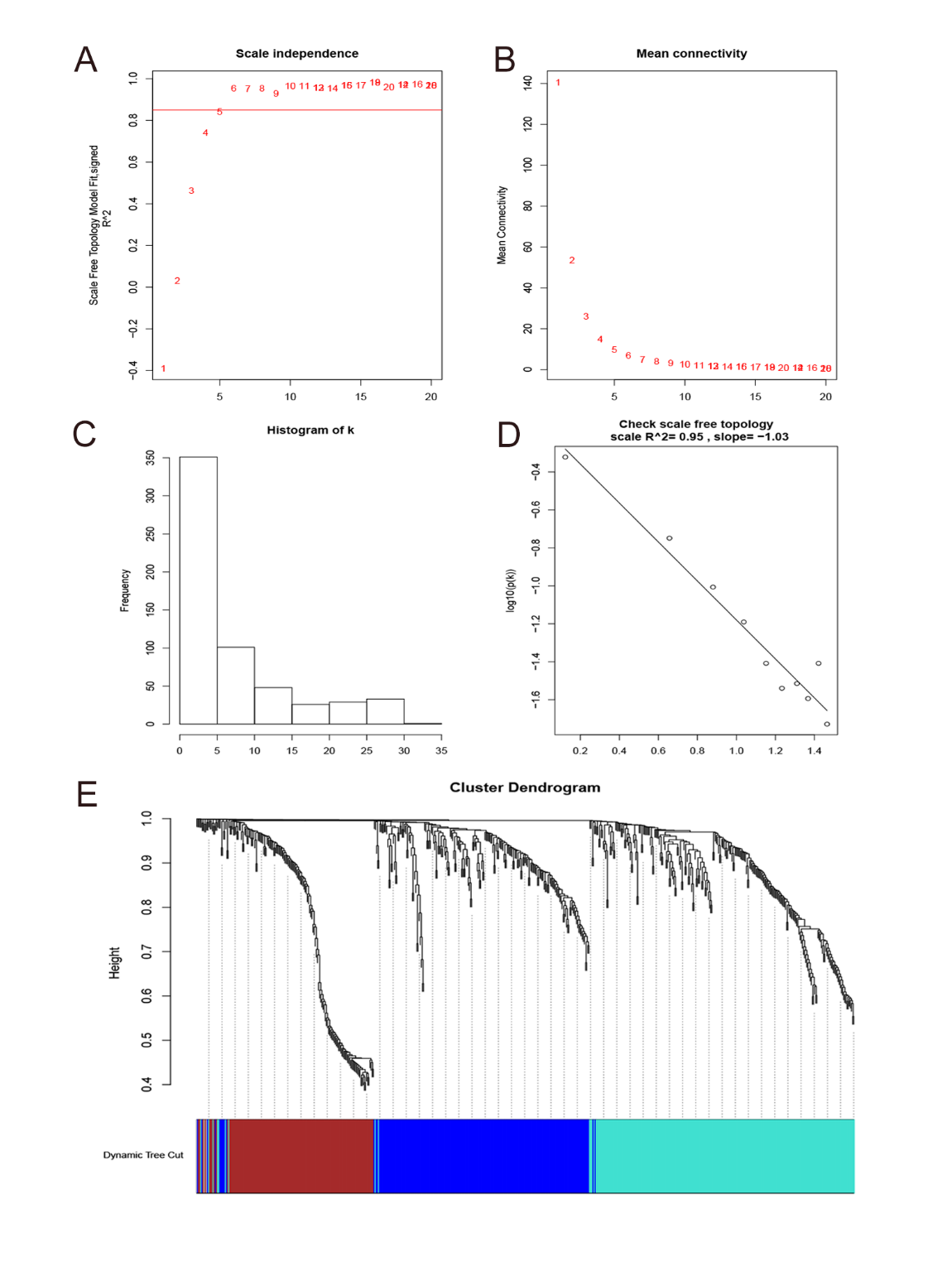


**Supplementary Figure S2. Determination of soft-thresholding power in the weighted gene co-expression network analysis (WGCNA). (A)** Analysis of the scale-free fit index for various soft-thresholding powers (β). **(B)** Analysis of the mean connectivity for various soft-thresholding powers. **(C)** Histogram of connectivity distribution when β = 6. **(D)** Checking the scale-free topology when β = 6. **(E)** Dendrogram of all differentially expressed genes clustered based on a dissimilarity measure (1-TOM).

**
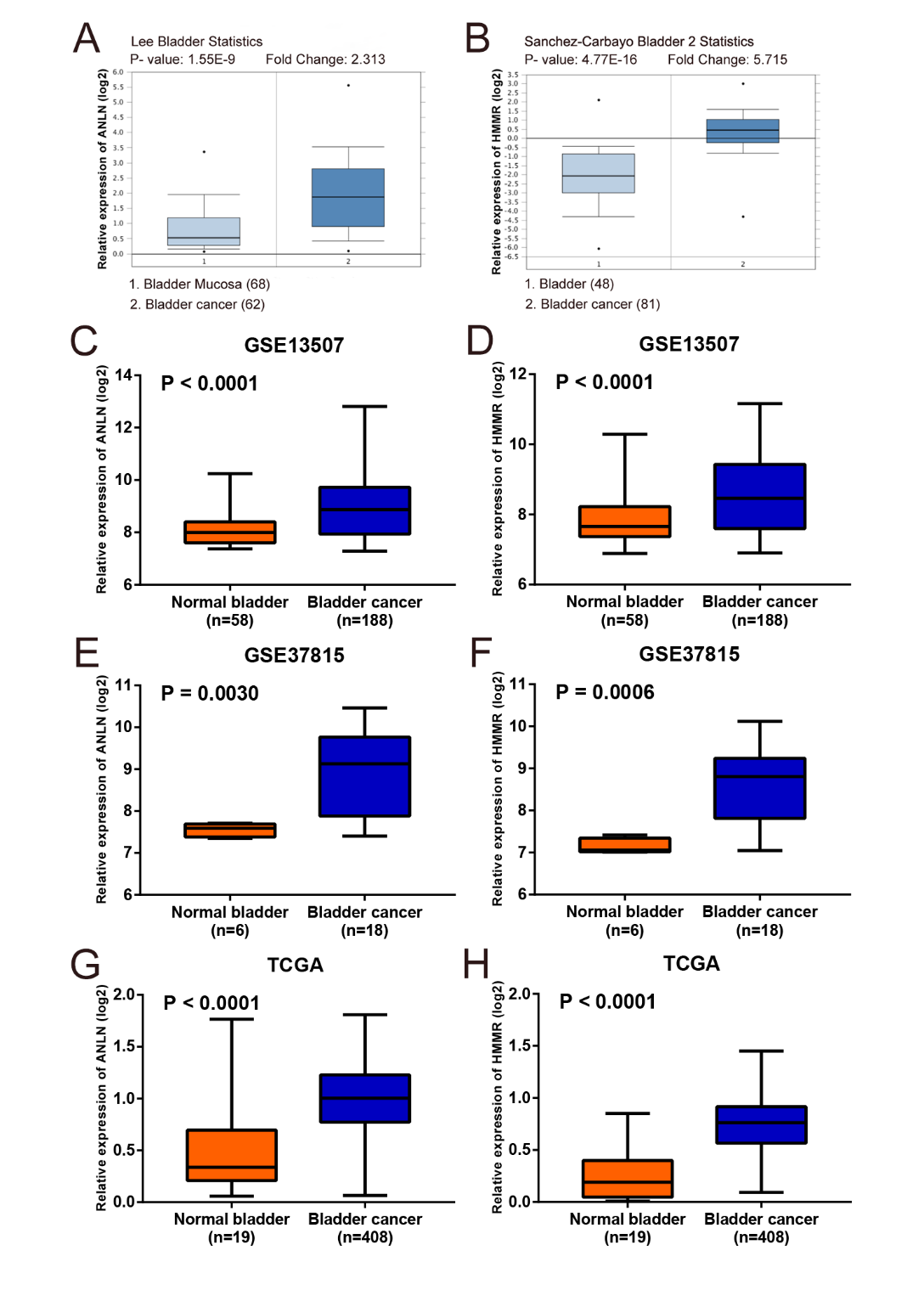
**

**Supplementary Figure S3.** Expression of hub gene between bladder cancer and normal bladder in Oncomine dataset (A, B), GSE13507 (C, D), GSE37815 (E, F) and TCGA dataset (G, H).

**
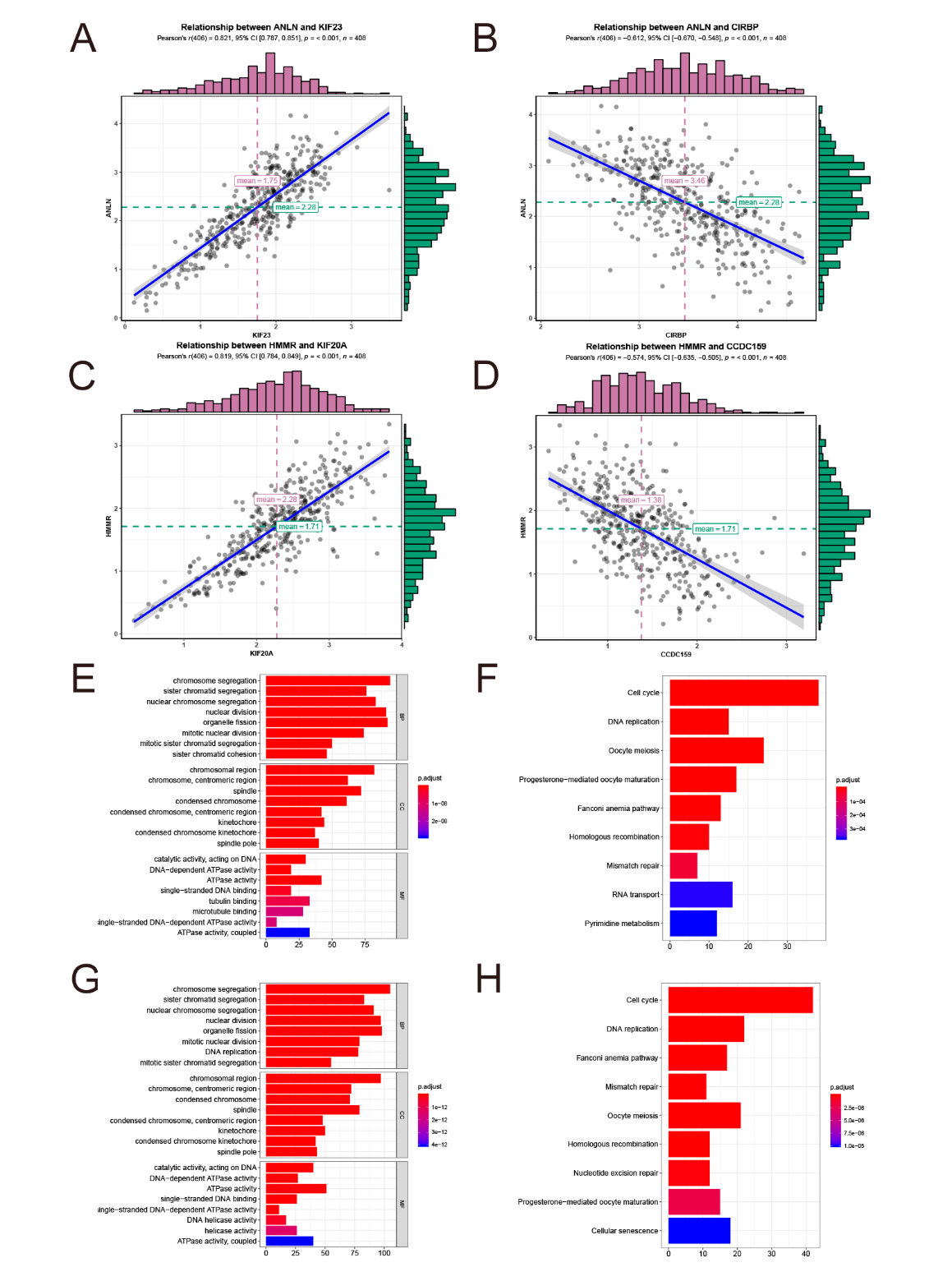
**

**Supplementary Figure S4. The correlation analysis of two hub genes.** The genes with the highest positive (A) and negative correlation (B) with ANLN were KIF23 and CIRBP; the genes with the highest positive (C) and negative correlation (D) with HMMR were KIF20A and CCDC159. The functional annotation of ANLN (E-F) and HMMR (G-H).

**
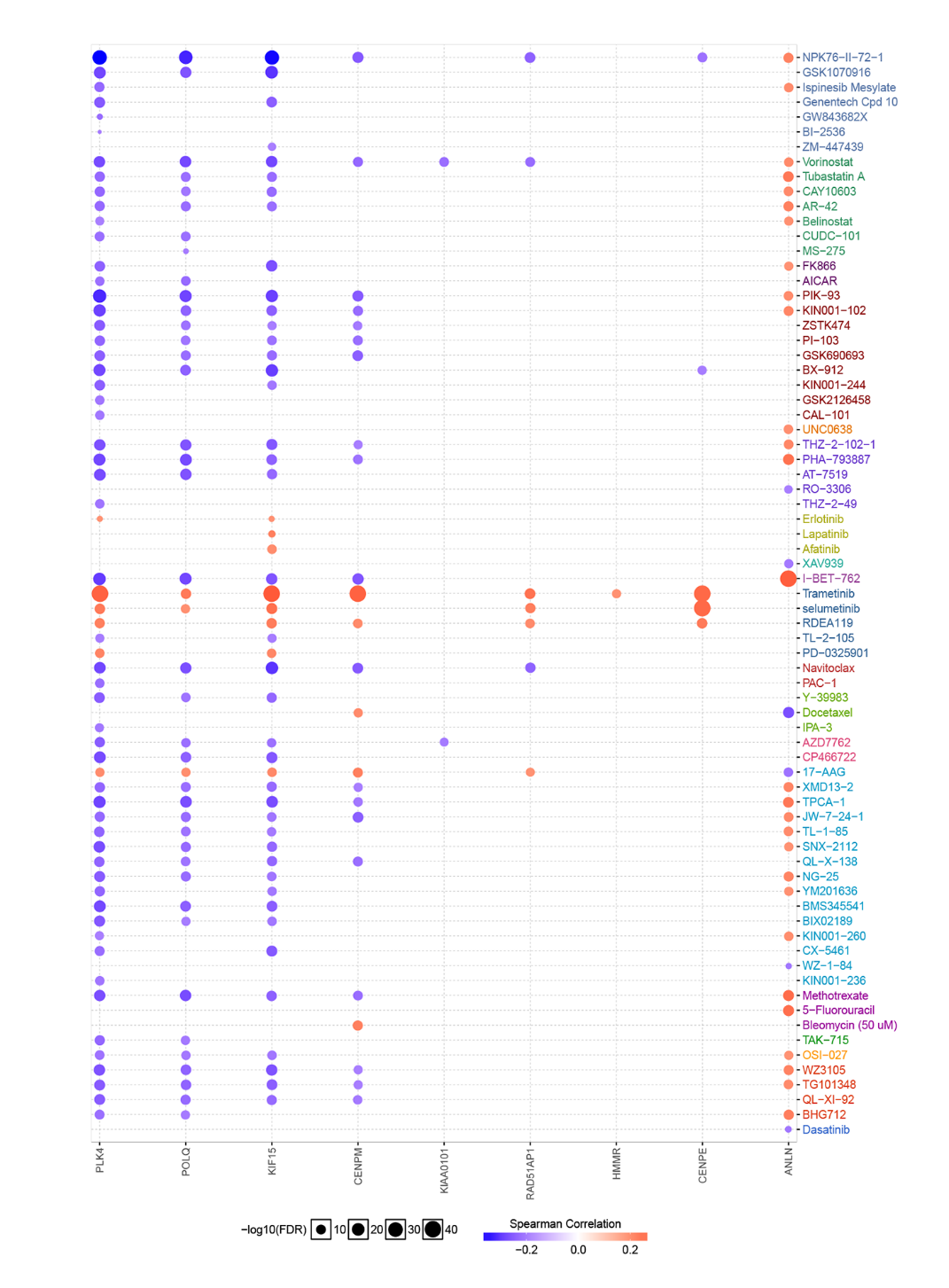
**

**Supplementary Figure S5. Sensitivity of hub genes in various drugs.** The red dots represent sensitivity to the drug, while blue dots represent the opposite.


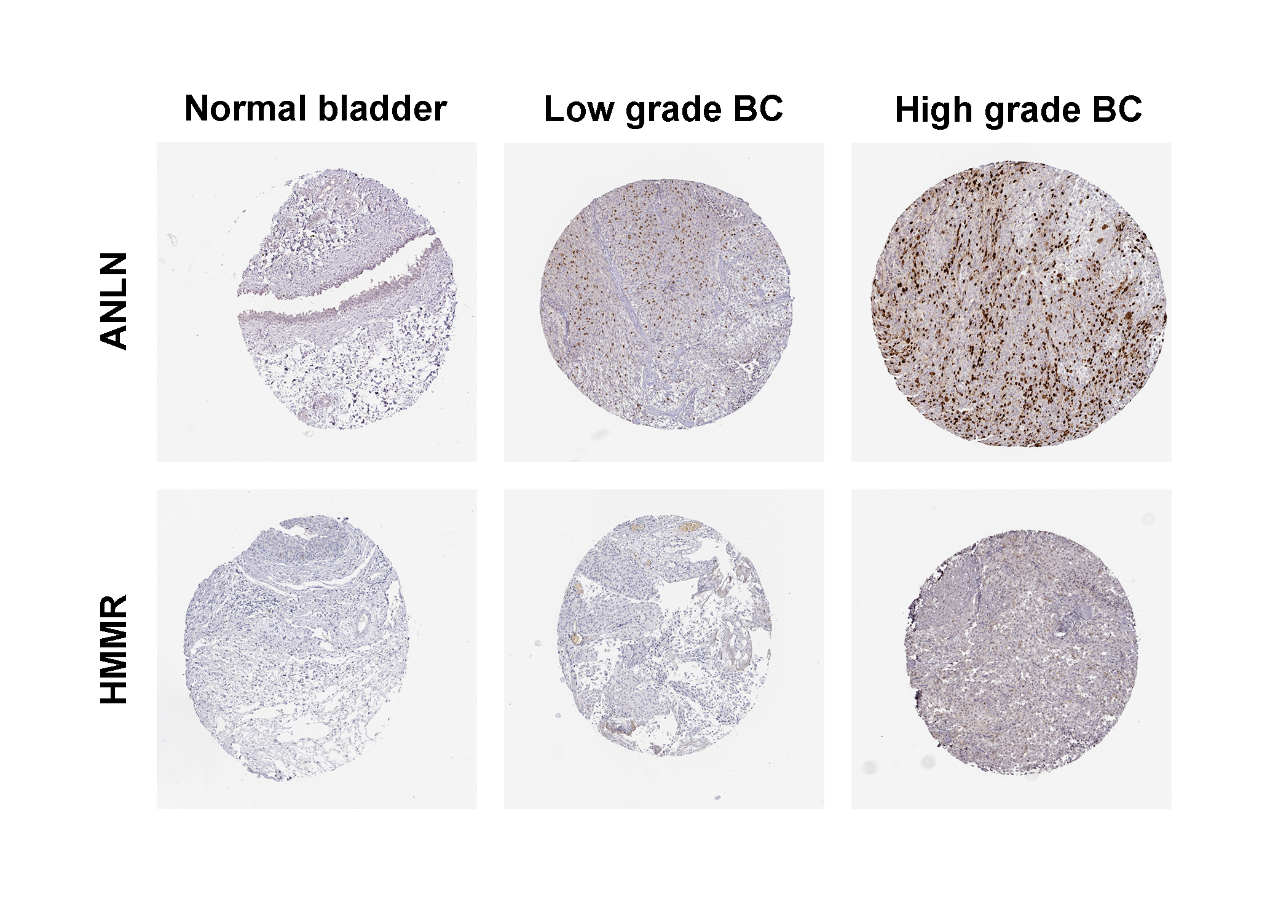


**Supplementary Figure S6.** verified the expression patterns of the protein levels of ANLN and HMMR in tissues in the HPA database.
